# Experimental Study and Mechanism Analysis of Paraffin/Sisal Composite Phase Change Energy Storage Fiber Prepared by Vacuum Adsorption Method

**DOI:** 10.3390/ma17020467

**Published:** 2024-01-18

**Authors:** Chun Chen, Qi Fu, Ruilin Cao, Zhenzhong Chen, Zedi Zhang, Kailun Xia, Nanqiao You, Yifan Jiang, Yamei Zhang

**Affiliations:** 1 Jiangsu Key Laboratory of Construction Materials, School of Materials Science and Engineering, Southeast University, Nanjing 211189, China; 101005550@seu.edu.cn (C.C.); 17775019206@163.com (Q.F.);; 2 School of Civil Engineering, Suzhou University of Science and Technology, Suzhou 215009, China

**Keywords:** sisal fiber, vacuum adsorption, composite phase change energy storage fiber, load ratio, storage mechanism

## Abstract

Sisal fiber exhibits a fibrous and porous structure with significant surface roughness, making it highly suitable for storing phase change materials (PCMs). Its intricate morphology further aids in mitigating the risk of PCM leakage. This research successfully employs vacuum adsorption to encapsulate paraffin within sisal fiber, yielding a potentially cost-effective, durable, and environmentally friendly phase change energy storage medium. A systematic investigation was carried out to evaluate the effects of sisal-to-paraffin mass ratio, fiber length, vacuum level, and negative pressure duration on the loading rate of paraffin. The experimental results demonstrate that a paraffin loading rate of 8 wt% can be achieved by subjecting a 3 mm sisal fiber to vacuum adsorption with 16 wt% paraffin for 1 h at −0.1 MPa. Through the utilization of nano-CT imaging enhancement technology, along with petrographic microscopy, this study elucidates the mechanism underlying paraffin storage within sisal fiber during vacuum adsorption. The observations reveal that a substantial portion of paraffin is primarily stored within the pores of the fiber, while a smaller quantity is firmly adsorbed onto its surface, thus yielding a durable phase change energy storage medium. The research findings contribute to both the theoretical foundations and the available practical guidance for the fabrication and implementation of paraffin/sisal fiber composite phase change energy storage mediums.

## 1. Introduction

Phase change materials (PCMs) experience transitions in response to alterations in the surrounding temperature, effectively either absorbing heat from, or releasing stored thermal energy into, the environment [1,2]. During latent heat storage, PCMs exhibit near-isothermal characteristics with high energy density. Paraffin, an organic PCM in a solid–liquid state, holds considerable potential due to its low cost, nontoxicity, low chemical reactivity, noncorrosiveness, and high plasticity. It has found extensive uses in various fields, including solar energy storage [3,4], building insulation [5,6], industrial waste heat recovery [7,8], the biomedical sector [9,10,11], and smart textiles [12,13]. Material leakage [14] and cost [15] currently are the primary constraints on PCM applications. Encapsulation technology, which entails enclosing PCMs within supporting materials to create shape-stable composite PCMs, represents one of the most effective approaches to addressing leakage concerns. Simultaneously, encapsulation technology significantly influences the overall cost of PCMs. According to market standards, nonencapsulated PCMs are only 1/7th the cost of encapsulated PCMs [16]. Hence, there is a pressing requirement to develop novel supporting materials and processes that can yield stable, cost-effective, and environmentally friendly composite PCMs.

Phase change microcapsules are widely recognized as an effective encapsulation method due to their high encapsulation efficiency and excellent leakage resistance. Nevertheless, the extensive use of phase change microcapsules has been impeded by their high production costs, preparation challenges, and limited thermal conductivity [17,18,19]. Furthermore, the utilization of phase change microcapsules is also associated with the risk of shell dissolution [20] or cracking [21,22,23] during operation. Using fibers to store phase change material is an innovative encapsulation technique in which PCM is combined with polymer matrices to produce heat storage and temperature-regulating functional fibers. The fiber matrix not only provides support and containment for the internal PCM but also enhances the thermal exchange efficiency with the external environment due to its large surface area to volume ratio [24]. Balaji [25] utilized a vacuum impregnation technique to introduce PCM into dehydrated and dried lotus stalks. The resulting biocomposite material was then deliberately oriented within epoxy resin. Leveraging the significant adsorption capacity and high porosity of the hollow microtubes in kapok fibers, Song [26] utilized them as carriers for confined encapsulation. The loading capacity of PCM within the microtubes had the potential to surpass 85 wt%. Moreover, a novel heat transfer pathway was developed through the uniform dispersion of high thermal conductivity nanoparticles on the fiber surface. Nevertheless, the prepared composite material displayed agglomeration, indicating a requirement for enhanced dispersibility. Electrospinning is the primary method for fabricating these fibers, involving the simultaneous dissolution of PCMs and supporting materials in organic solvents, leading to significant solvent usage and environmental concerns [27]. Moreover, electrospinning equipment can be costly, and the process itself is intricate. Adsorption technology, another prevalent encapsulation method, frequently utilizes natural or synthetic porous materials, such as expanded perlite [14,28], expanded vermiculite [29,30,31,32], as well as others [33,34,35,36,37], as supporting materials. In comparison to microcapsule and electrospinning technologies, adsorption technology exhibits superior cost-effectiveness and lower complexity. Nonetheless, the desorption of PCM poses a challenge that restricts its application [38]. Therefore, the advancement of supporting materials with increased loading stability will facilitate the expansion of application areas for PCMs.

The loading capacity of PCMs is primarily influenced by the pore structure of the supporting materials. Extensive research has been conducted to enhance the pore structure of supporting materials, aiming to improve both the loading efficiency and phase change enthalpy. Nomura [39] has indicated that pores larger than 10 μm are unsuitable for PCM retention, while excessively small pore sizes can modify the phase change behavior of materials. Gao [40] highlighted that pore sizes below 20 nm in supporting materials hinder the solidification of certain PCMs, resulting in a considerable decrease in latent heat values. According to Feng [41], for the PCM lauric acid in carbon nanotubes, as its loading ratio decreases the molecules tend to accumulate along the walls of carbon nanotube pores, creating a liquid layer that impedes the crystallization process within the pore centers. Dimberu [42] discovered for organic PCMs that the concentration of 3D porous metal supramolecular gels has a substantial impact on the specific surface area, pore size distribution, and characteristics, thereby facilitating the adjustment of the loading capacity. Considering the influence of supporting pore size on the adsorption and phase change behavior of PCMs, it is recommended to maintain the supporting material pore size within the range of 0.02–10 μm.

Wood fibers, obtained from abundant renewable resources, like kapok, sisal, and poplar, possess attributes such as biodegradability and affordability [43,44]. Sisal fiber, a prevalent variety among woody fibers, grows naturally in subtropical regions spanning the Americas, Africa, Asia, Oceania, and others. It exhibits advantages such as high strength, high elastic modulus, a large specific surface area, and resistance to wear and corrosion [45,46]. According to Huang [47], an analysis of sisal fiber’s economic costs reveals that its price is approximately 25% that of glass fiber and roughly 12% that of polypropylene fiber. In contrast to synthetic fibers, sisal fiber demonstrates minimal price fluctuations, wide availability, and environmentally friendly production processes. Regarding processing technology, the equipment used for sisal fiber treatment, mechanical cutting, and uniform mixing consists of standard instruments, thus reducing the environmental impact of the manufacturing process. The chosen sisal fiber for this study is priced at CNY 10 per kilogram, rendering it cost-effective. Extracted from naturally grown plants, it ensures sustainability and does not cause environmental pollution. Consequently, sisal fiber offers outstanding economic advantages and environmental friendliness, aligning with the principles of sustainability. Sisal fibers possess a microtubular structure characterized by an average outer diameter of around 200 μm. A cross-section of one sisal fiber contains around 100–200 conduits, with most conduits having a nearly polygonal or oval shape [48]. Currently, there is significant attention on natural adsorbents derived from sisal fibers due to their low density, high porosity, and excellent oil-absorbing properties [49]. The filamentous and porous nature of sisal fibers, coupled with their relatively high surface roughness, offers storage capacity for PCMs, potentially addressing challenges like PCM encapsulation difficulties and the high costs associated with encapsulation. In this study, PCM is securely stored within the pores and on the surfaces of sisal fibers, minimizing PCM leakage and contributing to environmental protection.

This study employed sisal fiber as the supporting material and utilized a vacuum adsorption method to impregnate the PCM (paraffin) into the three-dimensional pores of sisal fibers. This process resulted in the production of paraffin/sisal fiber composite phase change energy storage fibers, which we will refer to as ‘composite phase change fibers’ hereafter. The loading capacity of paraffin (hereafter referred to as ‘paraffin loading rate’) was further enhanced by adjusting the mass ratio of paraffin to sisal fibers, sisal fiber length, vacuum level, and negative pressure time. Nano-CT imaging enhancement technology and rock-phase microscopy were employed to observe and calculate the primary storage mechanism of paraffin within sisal fibers, as well as the mechanisms responsible for vacuum adsorption.

## 2. Materials and Methods

### 2.1. Materials

#### 2.1.1. Sisal Fiber

**Figure 1:**
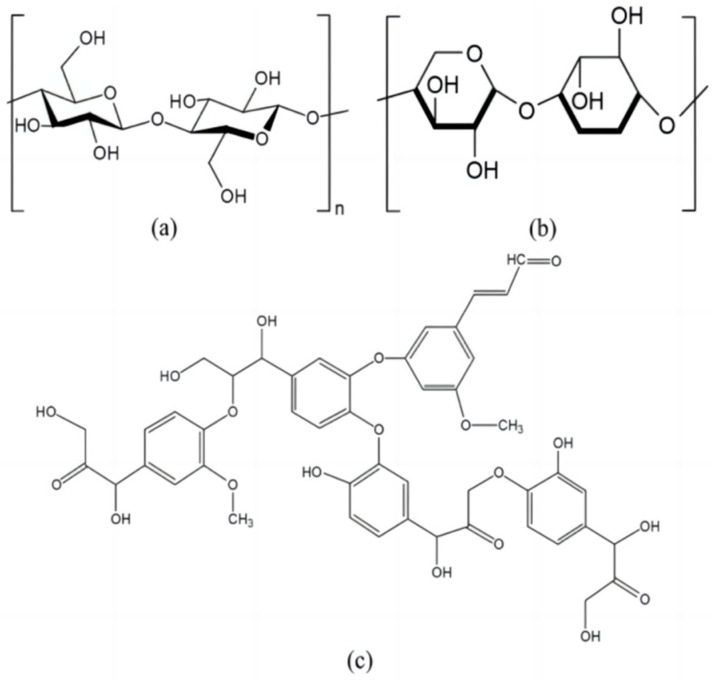
Chemical structure of the main chemical constituents of sisal fibers: (**a**) cellulose; (**b**) hemicellulose; and (**c**) lignin [52].

Table 1 and Table 2 provide details on the fundamental physical properties and chemical composition of sisal fiber, supplied by Guangxi Sisal Company (Nanning, China). Additionally, Figure 1 visually depicts its molecular structure.

**Table 1:** Physical properties of sisal fibers [50].

Density (g/cm^3^)	Fiber Diameter (μm)	Tensile Strength (MPa)	Elastic Modulus (GPa)	Rupture Elongation (%)	Stiffness (KN/mm)	Hygroscopicity (%)
1.47	150–300	470–720	17–22	2–5	30–38	11

**Table 2:** Chemical composition of sisal fibers [51].

Cellulose (wt%)	Lignin (wt%)	Hemicellulose (wt%)	Pectin (wt%)	Wax (wt%)
67–78	8–11	10–15	10	2

In this study, sisal fibers were used as the supporting material and a vacuum adsorption method was employed to imbue the three-dimensional pores of sisal fibers with the PCM paraffin. This process led to the production of composite phase change fibers, which consist of paraffin embedded within sisal fibers and are utilized for energy storage. Multiple factors, such as the mass ratio of paraffin to sisal fibers, sisal fiber length, vacuum level, and negative pressure time, were considered in order to maximize the loading capacity of the paraffin. To observe and quantify the primary storage mechanism of paraffin within sisal fibers, as well as the mechanisms involved in vacuum adsorption, Nano-CT imaging enhancement technology and rock-phase microscopy were employed.

#### 2.1.2. Paraffin

The experiment employed paraffin as the PCM, with a chemical formula of C_17_H_36_. Table 3 provides an overview of the pertinent properties of paraffin wax, as supplied by the manufacturing firm Shengbang Plastic Raw Material Factory (Dongying, China).

#### 2.1.3. Nanosilicon Dioxide

For nano-CT image enhancement, a hydrophobic nanosilicon dioxide (model VK-SP30S) was employed in the experiment. The hydrophobic nanosilicon dioxide (model VK-SP30S) has a particle size of 30 nm and a density of 2.4 g/cm^3^. It is manufactured by Zhentai Nano Company (Hangzhou, China).

### 2.2. Specimen Preparation

Pretreatment of sisal fibers was conducted by cutting them into three different lengths: 3 mm, 6 mm, and 9 mm. The fibers were then soaked and rinsed multiple times in clean water to eliminate surface dust. Afterward, they were dried in a 60 °C oven for 24 h, sealed for storage, and cooled to room temperature.

This study, following the preparation method detailed in Table 4, aimed to investigate the influence of sisal fiber lengths (3 mm, 6 mm, and 9 mm), paraffin-to-sisal fiber mass ratios (0%, 4%, 8%, 12%, 16%, and 20%), vacuum levels (atmospheric pressure of −0.05 MPa and −0.1 MPa), and vacuum exposure times (1 h, 2 h, and 3 h) on the loading capacity of paraffin within sisal fibers. The temperature of the vacuum apparatus was maintained at 50 °C throughout the preparation process.

**Figure 2:**
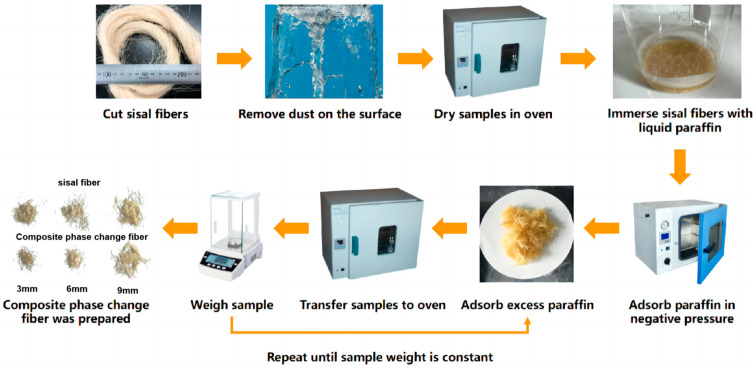
Preparation of paraffin/sisal fiber composite phase change fiber.

The postprocessing of the composite phase change fibers, prepared according to Table 4, entailed the removal of excess paraffin followed by the evaluation of the paraffin loading rate. The postprocessing procedure was as follows: the composite phase change fibers were cooled to room temperature, extracted, uniformly spread on filter paper, and transferred to a 60 °C oven. At each hour interval, the fibers were retrieved, cooled to room temperature, and weighed to determine their mass. The filter paper was replaced, and this process was repeated until the mass of the composite phase change fibers reached a stable state. The overall production process of the composite phase change fibers is depicted in Figure 2.

### 2.3. Experimental Procedure

#### 2.3.1. Petrographic Microscope Observation

The composite phase change fiber samples were placed on a glass slide for examination. An Axio Scope A1 polarizing microscope (Jena, Germany) was utilized to analyze the thickness and distribution of the paraffin layer on the surface of the sisal fibers.

#### 2.3.2. Three-Dimensional X-ray Microscopy

The sisal fiber pore morphology was investigated through three-dimensional scanning and reconstruction. The distribution of paraffin within the sisal fibers was examined using ZEISS Xradia 510 Versa nano-CT (Jena, Germany). The scanning parameters included a voltage of 50 kV, a current of 79 μA, and a CT resolution of dx = 7.63 μm, dy = 2.97 μm, and dz = 3.27 μm. Scans were performed at intervals of 1.5 μm along the length direction of the composite phase change fibers.

As indicated in Table 3, the density of paraffin is 0.76 g/cm^3^, which closely resembles the pores of sisal fibers observed in the nano-CT images, making direct observation challenging. Consequently, an appropriate contrast agent needs to be selected. Based on the principles of nano-CT imaging [53], substances with higher densities possess higher X-ray absorption coefficients. Hence, a contrast agent with a significantly higher density than paraffin, capable of being uniformly mixed, must be chosen. In the nano-CT images of the composite phase change fibers prepared with this contrast agent-added paraffin, a noticeable grayscale difference should be present. However, commonly used contrast agents like cesium chloride [54], barium chloride [55], and potassium iodide [56] exhibit poor solubility with paraffin. Additionally, the larger crystal particles of these agents face difficulties when entering the sisal fiber pores alongside the liquid paraffin.

In this study, nanosilica was chosen as the contrast agent, representing an innovative approach. The selected nanosilica possesses a density of 2.4 g/cm^3^, surpassing that of sisal fibers and pores. Furthermore, with a particle size of 30 nm, it is considerably smaller than the pore size of sisal fiber conduits, enabling it to penetrate the pores together with the liquid paraffin. Consequently, nanosilica effectively enhances the visualization of paraffin distribution within sisal fibers. The specific steps were as follows: 3 wt% of nanosilica was added to the test tube containing the liquid paraffin, which was then positioned in a KH-50B ultrasonic cleaner filled with hot water at 40 °C. Ultrasonic cleaning was carried out at a power of 50 W and a frequency of 40 KHz for a duration of 3 min to facilitate even dispersion of the nanosilica within the liquid paraffin.

## 3. Results and Discussion

The results of the paraffin loading rate for each group of the composite phase change fibers are presented in Table 5, compiled from the data available in Table 4. From the results in Table 5, it is evident that, in contrast to the first group (blank group), the remaining 15 groups consistently demonstrate the presence of stored paraffin in the composite phase change fibers, even after undergoing multiple heating cycles and paper adsorption. This observation indicates the capability of sisal fibers for stable retention of a certain amount of paraffin. Furthermore, Figure 2 illustrates that, in contrast to the findings of Song [26], the composite phase change fibers fabricated in this study exhibit independent existence, devoid of mutual adhesion, and manifest a more favorable state of dispersion.

**Figure 3:**
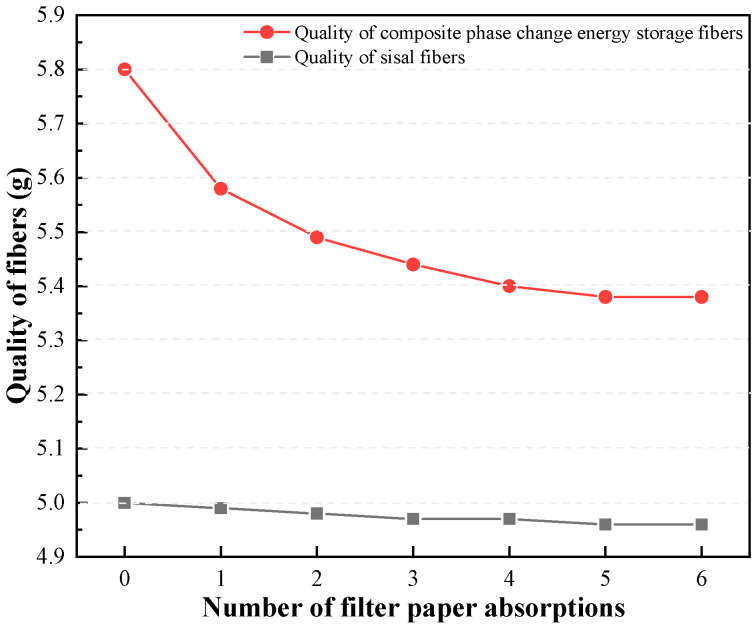
Changes in mass of sisal fibers and composite phase change fibers during cyclic heating process.

The postprocessing of the composite phase change fibers involved a method which utilized temperature cycling and paper adsorption to eliminate excess paraffin from the fibers. This ensured that the measured paraffin loading rate accurately represents the mass percentage of paraffin stably retained within the sisal fibers. To determine the optimal removal process parameters, an experimental study was conducted to examine the impact of the number of paper adsorption cycles on the effectiveness of paraffin removal. Taking the 1# and 5# test groups from Table 4 as examples, Figure 3 illustrates the changes in the mass of both the sisal fibers and the composite phase change fibers during the repetitive temperature cycling and paper adsorption procedure.

Based on Figure 3, it can be observed that both the cyclic heating and the number of paper adsorption cycles have negligible effects on the mass of the sisal fibers themselves. However, these processes do influence the mass loss of the composite phase change fibers to some extent. Initially, the mass loss rate of the composite phase change fibers increases rapidly with an increasing number of paper adsorption cycles, but eventually stabilizes. After undergoing six cycles of paper adsorption, the mass loss of the composite phase change fibers reaches a stable point. The paraffin loading rates presented in Table 5 were determined after six cycles of paper adsorption for all groups.

### 3.1. Influencing Factors and Changing Rules of Paraffin Loading Rate

#### 3.1.1. Effect of Mass Ratio between Paraffin and Sisal Fiber on Paraffin Loading Rate

**Figure 4:**
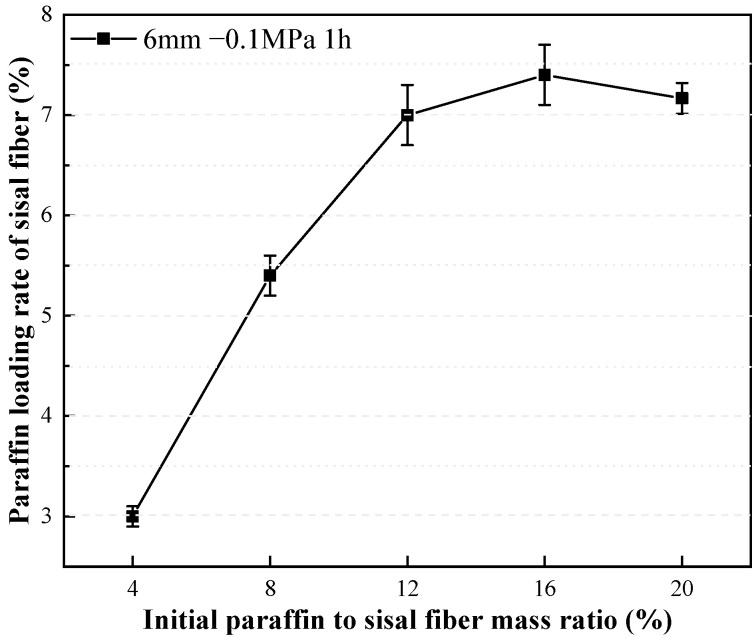
Effect of paraffin to sisal fiber mass ratio on paraffin loading rate.

The impact of the mass ratio of paraffin to sisal fibers on the paraffin loading rate is examined in Figure 4. It can be observed that with an increasing mass ratio of paraffin to sisal fibers, the rate of increase in the paraffin loading rate gradually slows down. Once the mass ratio of paraffin to sisal fibers reaches 16%, the maximum paraffin loading rate is achieved, indicating the saturation of the loading capacity of the sisal fibers. Further increasing the mass ratio of paraffin to sisal fibers will not enhance the paraffin loading rate, but instead result in the wastage of paraffin material that cannot be stably adsorbed by the sisal fibers. A detailed investigation into the storage mechanism of paraffin in sisal fibers is necessary to ascertain whether the paraffin is stored in the hollow conduits or on the surface of the sisal fibers. The different storage mechanisms ascertain the upper limit of sisal fibers’ capacity to store paraffin.

#### 3.1.2. Effect of Sisal Fiber Length on Paraffin Loading Rate

**Figure 5:**
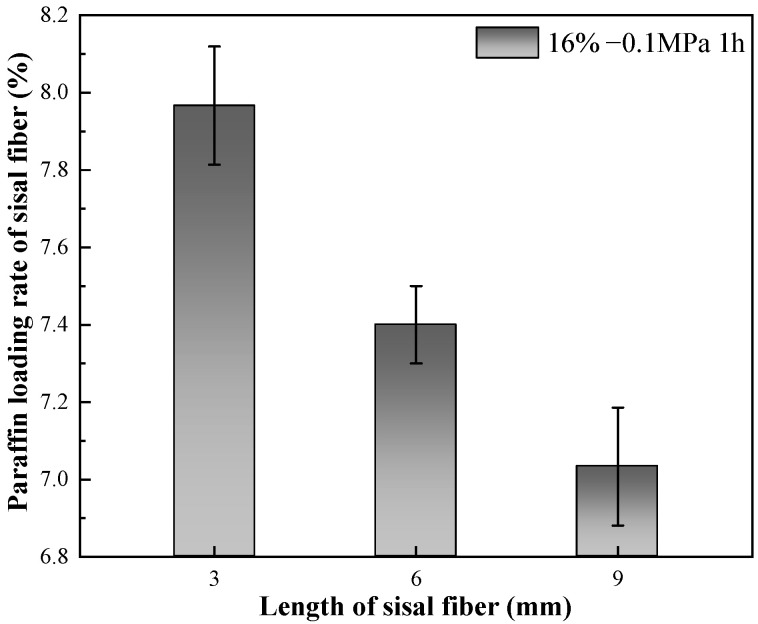
Effect of sisal fiber length on paraffin loading rate.

The impact of sisal fiber length on the paraffin loading rate is examined in Figure 5. As depicted in Figure 5, an increase in sisal fiber length results in a decrease in the maximum paraffin loading rate. This phenomenon can likely be attributed to the higher tendency of longer sisal fibers to interweave, leading to bending and obstruction of the internal pores. Consequently, the challenge of paraffin penetration increases. Additionally, the angles between bundled sisal fibers tend to aggregate paraffin, which can subsequently be adsorbed by the filter paper during the repeated heating process. Under consistent external negative pressure conditions, the capacity of negative pressure to facilitate the ingress of liquid paraffin into the sisal fiber pores remains constant. Nevertheless, the degree of sisal fiber pore expansion induced by negative pressure diminishes as sisal fiber length increases. As a result, the volume of paraffin penetrating the sisal fiber pores decreases with the rising fiber length, exhibiting a macroscopic reduction in paraffin loading rate as sisal fiber length increases. This observation is closely linked to the mechanism of paraffin storage in sisal fibers and the principles of vacuum adsorption.

#### 3.1.3. Effect of Vacuum Degree on Adsorption Rate of Paraffin

**Figure 6:**
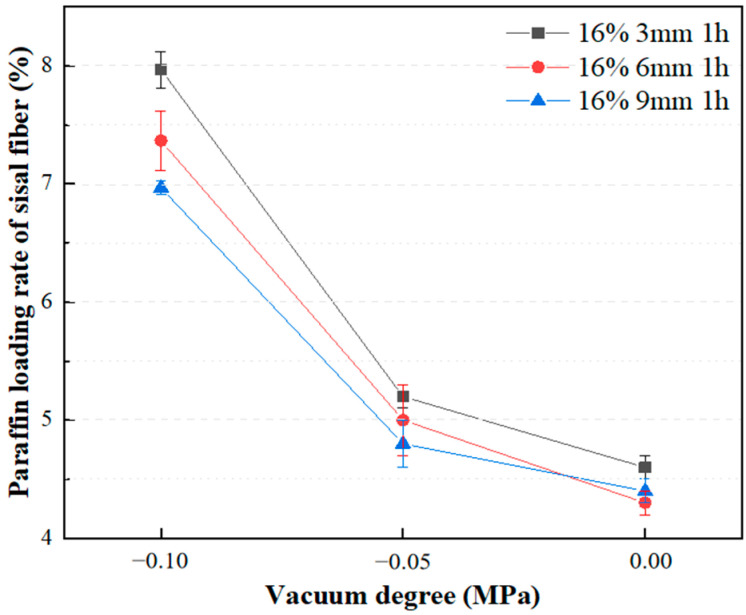
Effect of vacuum degree on paraffin loading rate.

Various intensities of vacuum negative pressure were applied to evaluate the paraffin loading rates of sisal fibers with different lengths, as shown in Figure 6. At atmospheric pressure, the maximum paraffin loading rates were similar for sisal fibers of different lengths. With increased vacuum levels, there were significant improvements in the paraffin loading rates. Under atmospheric pressure, the maximum paraffin loading rates were 4.4 wt%, 4.6 wt%, and 4.6 wt% for sisal fibers measuring 3 mm, 6 mm, and 9 mm, respectively. After 1 h of vacuum adsorption at −0.1 MPa, the maximum paraffin loading rates increased by 82%, 61%, and 52% for the corresponding fiber lengths. The application of vacuum negative pressure induces a pressure differential between the inside and outside of the fiber conduits, facilitating the injection of liquid PCMs into the conduits. Simultaneously, the presence of reserved volumes within the conduits prevents leakage issues of the PCMs stored inside, which can be caused by thermal expansion, external forces, and external compressions [26]. Moreover, based on the observations in Figure 6, it is evident that at a vacuum degree of 0, paraffin adsorption on 3 mm sisal fibers is 4.5% greater than on 9 mm sisal fibers. With a decrease in vacuum degree to −0.1 MPa, paraffin adsorption on 3 mm sisal fibers is 14.3% greater than on 9 mm sisal fibers. Comparatively, the rise in negative pressure intensity exerts a more pronounced influence on paraffin adsorption on sisal fibers than does the reduction in sisal fiber length.

#### 3.1.4. Effect of Negative Pressure Time on Paraffin Loading Rate

**Figure 7:**
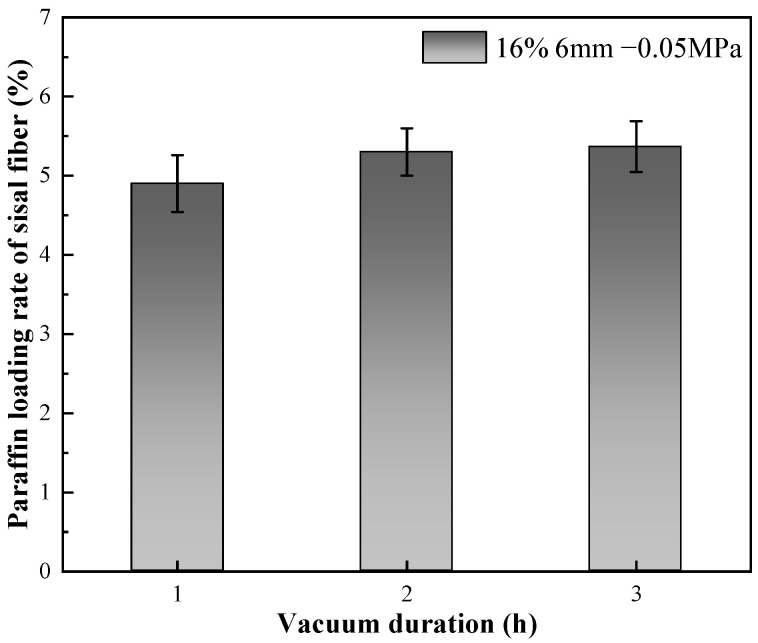
Impact of vacuum duration on paraffin loading rate.

The paraffin loading rate as a function of vacuum duration is illustrated in Figure 7. As depicted in the figure, there is no substantial increase in the paraffin loading rate with an extended vacuum duration. This observation implies that the adsorption process of sisal fiber with paraffin is relatively quick. The experimental setup for adsorption time has exceeded the saturation point [57], indicating that the adsorption process is rapidly completed without substantial improvement over time. However, further confirmation of this phenomenon is warranted through an in-depth analysis of the paraffin storage mechanism within sisal fiber.

### 3.2. Storage Mechanism and Vacuum Adsorption Mechanism of Paraffin in Sisal Fiber

The influence of vacuum negative pressure time on paraffin loading rates is depicted in Figure 7. From the figure, it is evident that prolonging the negative pressure time does not result in a significant increase in the paraffin loading rate. This implies that the adsorption process of paraffin by sisal fibers has a relatively short effective duration and quickly reaches completion without substantial improvement over time. Consequently, an extensive investigation is necessary to validate and elucidate the mechanism accountable for paraffin storage within sisal fibers.

#### 3.2.1. Experimental Study on the Storage Location of Paraffin in Sisal Fiber

**Figure 8:**
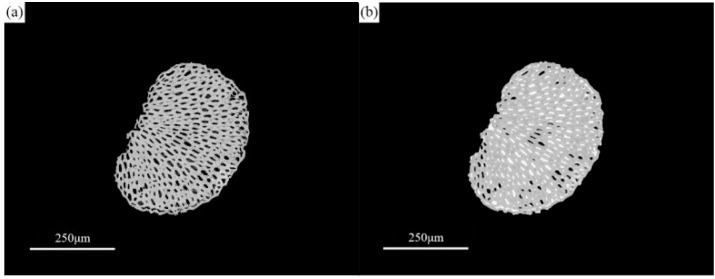
Nano-CT section images of (**a**) sisal fiber and (**b**) composite phase change fiber.

**Figure 9:**
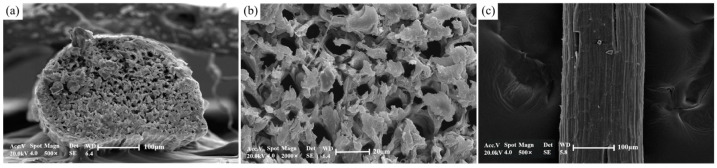
Morphology of sisal fibers observed by SEM: (**a**) cross-sectional view, (**b**) pores in the cross section observed at high magnification and (**c**) longitudinal view.

The investigation into the storage location of paraffin within sisal fibers involved a comparison of nano-CT slice images of the same sisal fiber before and after the paraffin composite formation. Figure 8a displays a slice image of the cross section of a sisal fiber prior to paraffin impregnation, revealing its mesh-like structure with numerous internal pores, corroborating Figure 9a,b. With formulation 7# from Table 4, paraffin containing nanosilica replaced the conventional paraffin, forming the composite phase change fiber in combination with the same sisal fiber. The composite was then subjected to three-dimensional scanning and reconstruction, resulting in a collection of 1992 cross sectional slices. Among these slices, those corresponding to the same position as Figure 8a were selected and are presented in Figure 8b. Since nanosilica served as the nano-CT image enhancer, the position of the paraffin containing nanosilica in the composite phase change fiber was indirectly characterized. A comparison between Figure 8a,b reveals three distinctive regions with different CT values (gray levels) in the composite phase change fiber: sisal fiber regions with gray values ranging from 110–143, pore areas without paraffin, with gray values between 82–102, and regions containing nanosilica, with gray values ranging from 145–183, which indirectly indicates the presence of paraffin components. These regions primarily reside on the surface of sisal fibers and within the majority of the pores. The nano-CT imaging method employed in this study, in contrast with the microscopic observation technique used in the research of Song [26], provides a more lucid and intuitive depiction of the storage locations and impregnation depth of PCMs within the fibers.

Using ImageJ 2 software, we measured the areas of sisal fibers and pores in Figure 8a. To determine the total porosity of sisal fibers, we calculated the ratio of the pore areas to the combined areas of sisal fibers and pores. In Figure 8b, we separately measured the areas of unfilled paraffin pores, paraffin containing nanosilica, and sisal fibers, to evaluate the filling effect of paraffin on the fibers. The results indicate that the total porosity of sisal fibers is 14%. Following vacuum adsorption of paraffin, approximately 88% of the pore area is filled with paraffin. This finding suggests that the sisal fiber conduits serve as the primary storage spaces for paraffin, and the total porosity of sisal fibers may represent the upper limit of the paraffin loading rate. Even under the current experimental conditions, where a substantial amount of paraffin is loaded, there remains reserved space within the cavities. This ensures that when the phase change material expands due to heating or the composite phase change fiber undergoes compression or deformation under stress, there is sufficient capacity to prevent paraffin from leaking out of the conduit openings [26,58]. The stable adsorption method effectively prevents environmental pollution caused by PCM leakage, showcasing the environmental friendliness of composite phase change fibers.

**Figure 10:**
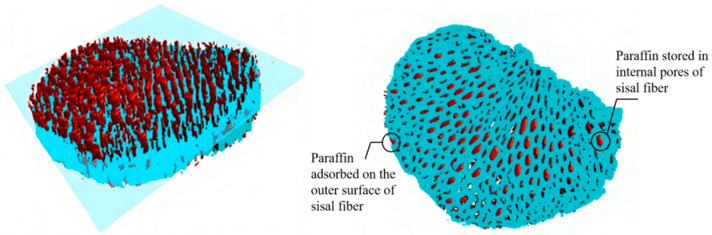
3D reconstruction of nano-CT in the middle section of composite phase change fibers (blue area is sisal fiber, red area is paraffin).

Figure 10 presents a three-dimensional reconstructed image captured from the middle section of the composite phase change fiber. The grayscale threshold range was adjusted to encompass the grayscale regions covering sisal fibers across all slices and the regions containing silicon dioxide paraffin. Dragonfly V2.0.9 3D visualization software was utilized for the reconstruction of the composite phase change fiber. The image in Figure 10 vividly demonstrates the storage of paraffin in the middle section of the composite phase change fiber. It is evident that the sisal fibers form a bundle of interconnected conduits with diameters ranging from 5–20 μm. The presence of open and interconnected pores within this diameter range facilitates stable retention of paraffin without interfering with the phase change process [40,43,59]. Liquid paraffin permeates from both ends of the sisal fiber. In Figure 10, the red columnar region represents the longitudinal distribution of paraffin along the sisal fiber conduits, devoid of any fractures or discontinuities. This implies that even in the middle section of the composite phase change fiber, paraffin effectively permeates the sisal fibers. Under specific vacuum adsorption conditions, the penetration depth of paraffin along the longitudinal axis of sisal fibers is nearly equal to the length of the fibers, enabling efficient utilization of the sisal fiber conduits as paraffin storage spaces.

The blue meshed area in Figure 10 illustrates the three-dimensional reconstruction of sisal fibers, while the red columnar region denotes the three-dimensional reconstruction of paraffin containing silicon dioxide. By considering the outer boundary of the blue area as a boundary, the red areas within and outside of this boundary represent paraffin stored in the internal pores of the sisal fibers and paraffin adsorbed on the outer surface of the sisal fibers. The volumes of these two components of paraffin in the composite phase change fiber were separately calculated using Dragonfly. The results of the calculations reveal that 93% of the paraffin is stored in the sisal fiber conduits, with only a minimal amount of paraffin adhering to the surface of the sisal fibers. This storage mechanism effectively reduces the risk of PCM leakage.

**Figure 11:**
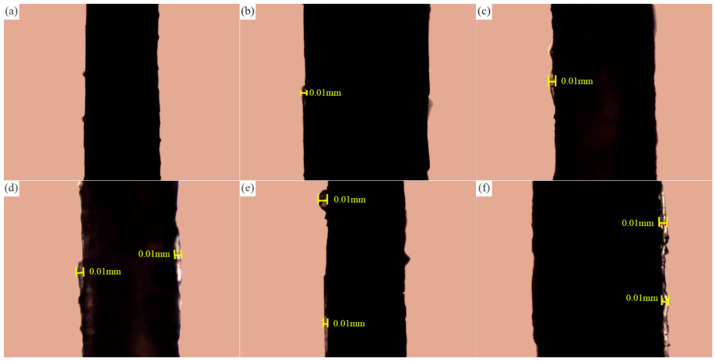
Variation of paraffin layer on the surface of sisal fibers with different paraffin to sisal fiber mass ratios: (**a**) 0%; (**b**) 4%; (**c**) 8%; (**d**) 12%; (**e**) 16%; and (**f**) 20%.

In order to visually examine the adsorption of paraffin on the surface of sisal fiber, composite phase change fibers labeled as 1#–6# in Table 4 were selected for observation under a petrographic microscope. Prior to paraffin loading, as depicted in Figure 9c and Figure 11a, the surface of the sisal fiber appeared relatively smooth, with occasional depressions or protrusions. Following vacuum adsorption of paraffin, the surface of the sisal fiber exhibited distinct layers of paraffin adsorption, as shown in Figure 11b–f. Figure 11 reveals a positive correlation between the mass ratio of paraffin to sisal fiber and the amount of paraffin stored on the surface of the fiber. This correlation is evident in the length and thickness of the areas covered by paraffin on the surface of the sisal fiber. The process of paraffin adsorption on the sisal fiber surface typically involves preferential adsorption and aggregation of paraffin at the uneven regions. As the mass ratio of paraffin to sisal fiber increases, this area accumulates more paraffin, resulting in increased coverage and thickness of the paraffin layer both longitudinally and laterally. However, upon surpassing a specific threshold, the weakening interaction forces between the paraffin and the surface of the sisal fibers, due to increased distance, hinder excess paraffin from stably adhering to the fibers’ surfaces. Experiments also revealed that the thickness of the paraffin adsorption layer did not significantly increase with higher mass ratios of paraffin to sisal fiber, with the thickness of the paraffin layer on the fiber surface not exceeding 0.01 mm. These findings suggest that while sisal fibers can store a small amount of paraffin through surface adsorption, it is not their primary storage mechanism.

#### 3.2.2. Vacuum Adsorption Mechanism

**Figure 12:**
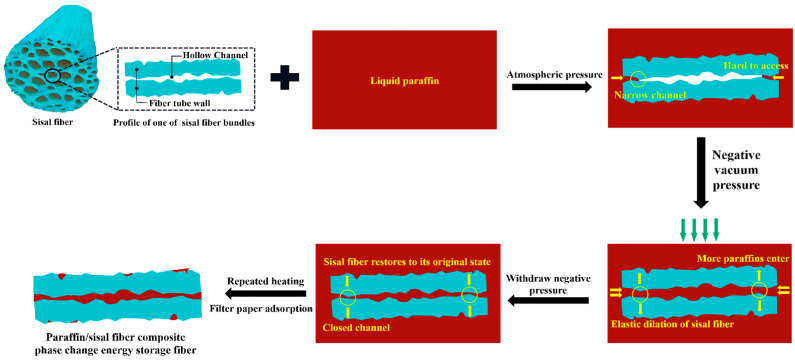
Schematic diagram of vacuum adsorption mechanism.

The results from Section 3.1.3 indicate a significant increase in paraffin loading with increased vacuum pressure. As analyzed in Section 3.2.1, sisal fibers primarily use hollow conduits to store paraffin. Therefore, changes in vacuum pressure mainly alter the internal pore spaces of the sisal fiber, subsequently affecting the ease and scope of paraffin infiltration. The vacuum adsorption mechanism is illustrated in Figure 12.

At atmospheric pressure, it is challenging for liquid paraffin to penetrate the narrower passages in the sisal fiber. Applying a vacuum generates negative pressure, expanding the hollow conduits within the sisal fiber and causing the narrower passages to open up. This creates a pressure difference between the exterior and interior, driving liquid paraffin to flow along the conduits and equalize the pressures. Even when the temperature exceeds the paraffin’s melting point, the sisal fiber effectively immobilizes the paraffin using capillary action, surface tension within the conduits, and other forces like hydrogen bonding [60]. Upon returning to normal pressure, the expansion of the hollow conduits in the sisal fiber reduces. The reserved volume within these conduits prevents paraffin leakage caused by thermal expansion and compression, achieving confined encapsulation [58]. Increasing the vacuum pressure leads to a greater expansion of the hollow conduits in the sisal fiber and a more thorough filling of paraffin along the conduit direction, resulting in increased paraffin loading. The consistent pattern of changes observed across different lengths of sisal fibers in Section 3.1.3 supports the confirmation of the vacuum adsorption mechanism.

The results from Section 3.1.3 reveal that shorter sisal fibers exhibit a more significant increase in the rate of paraffin loading at higher vacuum levels. This test result can be reasonably explained by the vacuum adsorption mechanism. As the sisal fibers become longer, it becomes more challenging to expand the conduits, requiring higher pressure for the liquid paraffin to penetrate. Therefore, achieving complete filling of longer sisal fiber conduits necessitates a higher vacuum level.

The experimental results indicate that the internal porosity of sisal fibers is the limiting factor for the paraffin loading rate. Simply improving the surface properties, such as by alkali treatment or fiber surface modification, is not very effective in enhancing the loading rate. To increase the internal porosity and expand the storage space for paraffin within the fiber pores, it is recommended to choose appropriate sisal fiber varieties, increase vacuum levels, or utilize techniques like vacuum cracking. These measures will enhance the loading rate of composite phase change fibers.

## 4. Conclusions

The study systematically examined the influence of factors such as the mass ratio of paraffin to sisal fiber, sisal fiber length, vacuum level, and negative pressure time on the rate of paraffin loading. Based on the experimental results and in-depth analysis, the following conclusions can be drawn:

Through the implementation of vacuum impregnation, stable, cost-effective, and environmentally friendly composite phase change fibers were successfully prepared. The optimal experimental parameters, using the sisal fibers employed in this study, were determined to be a sisal fiber length of 3 mm and a paraffin-to-sisal fiber mass ratio of 16%. The paraffin loading rate can reach 8 wt% under a vacuum pressure of −0.1 MPa for 1 h.

1. Increasing the mass ratio of paraffin to sisal fibers leads to a decrease in the rate of paraffin loading by sisal fibers, indicating a limited capacity for paraffin storage within them. Vacuum adsorption allows paraffin to impregnate the internal areas of sisal fibers, which are typically inaccessible under normal pressure, thereby increasing the storage space and enhancing the loading rate. The efficiency of vacuum adsorption depends on the vacuum level and the length of the sisal fibers. Higher vacuum levels are advantageous for increasing the paraffin loading rate. However, longer sisal fibers exhibit a lower increase in loading rate compared to shorter fibers at the same vacuum level. The influence of vacuum degree on the paraffin loading rate surpasses the impact of fiber length on the paraffin loading rate. Importantly, extending the duration of vacuum adsorption does not effectively improve the paraffin loading rate. Their relatively low loading capacity may be offset by using more fibers. These composite phase change fibers also exhibit excellent strength enhancement effects, and further work will enable a more thorough quantitative assessment of their thermal performance in applications such as building insulation, in particular their cost-effectiveness and long-term stability.
2. By utilizing nano-CT and image processing techniques, the composite phase change fibers were reconstructed in three dimensions. These observations were corroborated with results from petrographic microscopy. The study confirms the potential of the fibrous porous structure and rough surface of sisal fibers to serve as storage spaces for PCMs, with a primary focus on the internal pores of sisal fibers. The mechanism of internal pore storage, combined with the ideal mechanical strength of these fibers and the presence of reserved spaces, enables the microscopic solid–liquid phase change of paraffin while maintaining the macroscopic integrity of the composite phase change fibers in a fully solid state. This mechanism ensures the structural stability of the material, even under compression and stress, and reduces the risk of paraffin leakage.

## Figures and Tables

**Table 3:** Physical properties of phase change paraffins.

Phase Transition Temperature (°C)	Solid Density (kg/L)	Liquid Density (kg/L)	Enthalpy (kJ/kg)	Specific Heat Capacity (J/(g·K))	Thermal Conductivity (W/(m·K))	Flash Point (°C)
25 ± 2	0.76	0.86	≥140	2.14	0.21	≥110

**Table 4:** Preparation scheme of composite phase change fibers.

Groups	Sisal Fiber (g)	Sisal Fiber Length (mm)	Paraffin (g)	Paraffin to Sisal Fiber Mass Ratio (%)	Vacuum Degree (MPa)	Vacuum Duration (h)
1#	5	6	0	0	−0.1	1
3#	5	6	0.4	8	−0.1	1
4#	5	6	0.6	12	−0.1	1
5#	5	6	0.8	16	−0.1	1
6#	5	6	1.0	20	−0.1	1
7#	5	3	0.8	16	−0.1	1
8#	5	9	0.8	16	−0.1	1
9#	5	3	0.8	16	0	1
10#	5	6	0.8	16	0	1
11#	5	9	0.8	16	0	1
12#	5	3	0.8	16	−0.05	1
13#	5	9	0.8	16	−0.05	1
14#	5	6	0.8	16	−0.05	1
15#	5	6	0.8	16	−0.05	2
16#	5	6	0.8	16	−0.05	3

**Table 5:** Paraffin loading rate of different groups of composite phase change fibers.

Groups	Paraffin Loading Rate (wt%)	Groups	Paraffin Loading Rate (wt%)
1#	0	9#	4.4
2#	3	10#	4.4
3#	5.4	11#	4.6
4#	7	12#	5
5#	7.4	13#	5.2
6#	7.2	14#	4.8
7#	8	15#	5.6
8#	7	16#	5.6

## Data Availability

Data will be made available on request.
